# DNA moves sequentially towards the nuclear matrix during DNA replication in vivo

**DOI:** 10.1186/1471-2121-12-3

**Published:** 2011-01-19

**Authors:** Juan Carlos Rivera-Mulia, Rolando Hernández-Muñoz, Federico Martínez, Armando Aranda-Anzaldo

**Affiliations:** 1 Laboratorio de Biología Molecular, Facultad de Medicina, Universidad Autónoma del Estado de México, Apartado Postal 428, C.P. 50000, Toluca, Edo. Méx., México; 2 Departamento de Biología Celular, Instituto de Fisiología Celular, UNAM, Apartado Postal 70-243, C.P. 04510, México D.F., México; 3 Departamento de Bioquímica, Facultad de Medicina, UNAM, Apartado Postal 70-159, C.P. 04510, México D.F

## Abstract

**Background:**

In the interphase nucleus of metazoan cells DNA is organized in supercoiled loops anchored to a nuclear matrix (NM). There is varied evidence indicating that DNA replication occurs in replication factories organized upon the NM and that DNA loops may correspond to the actual replicons in vivo. In normal rat liver the hepatocytes are arrested in G0 but they synchronously re-enter the cell cycle after partial-hepatectomy leading to liver regeneration in vivo. We have previously determined in quiescent rat hepatocytes that a 162 kbp genomic region containing members of the albumin gene family is organized into five structural DNA loops.

**Results:**

In the present work we tracked down the movement relative to the NM of DNA sequences located at different points within such five structural DNA loops during the S phase and after the return to cellular quiescence during liver regeneration. Our results indicate that looped DNA moves sequentially towards the NM during replication and then returns to its original position in newly quiescent cells, once the liver regeneration has been achieved.

**Conclusions:**

Looped DNA moves in a sequential fashion, as if reeled in, towards the NM during DNA replication in vivo thus supporting the notion that the DNA template is pulled progressively towards the replication factories on the NM so as to be replicated. These results provide further evidence that the structural DNA loops correspond to the actual replicons in vivo.

## Background

In the interphase nucleus of metazoan cells DNA is organized in supercoiled loops anchored to a substructure commonly known as the nuclear matrix (NM). The NM is obtained by extracting the cells in presence of high-salt, non-ionic detergents and DNase and its specific composition is still a matter of debate as some four hundred proteins have been associated with such a substructure [1-4]. DNA is attached to the NM by non-coding sequences known as matrix attachment regions or MARs. So far there are no specific consensus sequences for a priori defining a MAR although most well-characterized MARs are rich in A-T [5]. Very few specific MAR-binding proteins have been characterized so far [6] but the large number of DNA loops in a given cell suggests that DNA-NM interactions occur on a grand scale by means of so-called indirect readouts that do not depend on interactions between specific NM proteins and specific DNA sequences but more likely on the recognition of local DNA structure in 3D [7,8]. MARs have been operationally classified into structural-constitutive, resistant to high-salt extraction, and functional-facultative, non-resistant to high-salt extraction [9,10]. Therefore the resulting DNA loops can be also classified into structural and functional, and the MARs attaching the structural DNA loops to the NM are also known as loop anchorage regions or LARs [9]. For some time it has been speculated that DNA loops correspond to independent functional domains of chromatin. However, it has already been shown that a single transcriptional unit may be organized into several structural DNA loops [11] and further evidence suggests there is no correlation between the structural DNA loops and transcription units [10,12-14]. On the other hand, there is varied evidence suggesting that the structural DNA loops may correspond to the actual replicons [9]. Indeed, DNA replication occurs in mammalian cells at so-called replication foci or factories occupying defined nuclear sites at specific times during S phase [15] and there is important evidence that such replication factories are organized upon the NM [16-19]. Moreover, theoretical implications resulting from considering the topology of DNA and the actual size of the replication complexes that include enormous polymerizing machines that dwarf the DNA template, suggest that replication of mammalian DNA in vivo involves fixed polymerases in replication foci that reel in their templates as they extrude newly made DNA [20]. This coupled to varied experimental evidence suggests that the NM is the structural support of DNA replication [21].

The hepatocytes are cells that preserve a proliferating capacity that is elicited in vivo after partial ablation of the liver, leading to liver regeneration in experimental animals such as the rat. The previously quiescent G0 hepatocytes synchronously re-enter the cell cycle after partial hepatectomy [22]. Evidence obtained using this animal model suggested that the DNA replication sites are continuously bound to the NM leading to the proposal that DNA may replicate either by reeling through fixed replication complexes on the NM or that replication proceeds by sliding of the complexes through the loops until reaching the corresponding LARs [16]. We have previously shown that specific DNA sequences located in different chromosomes, thus representing several territories within the interphase nucleus, change their original position relative to the NM during liver regeneration, becoming quite proximal to the NM during the peak of DNA synthesis at 24 h after partial hepatectomy and then recover their original positions once the liver regeneration has been achieved and the hepatocytes return to quiescence [10,13]. Using a topological approach, we have determined in primary rat hepatocytes that a 162 kbp region that includes several genes of the albumin gene family is organized in vivo into five structural DNA loops [23]. In the present work we tracked down the movement relative to the NM of target DNA sequences located at different points within such five structural loops, during the process of liver regeneration. Our results suggest that looped DNA moves in a sequential fashion, as if reeled in, towards the NM (where the replication complexes are assembled) during DNA replication in vivo and then returns to its original position in newly quiescent cells, once the liver regeneration has been achieved, thus providing further evidence that the structural DNA loops correspond to the actual replicons in vivo.

## Results

### Kinetics of nucleoid-DNA digestion as a function of the DNA-replicating status of primary rat hepatocytes

**Figure 1:**
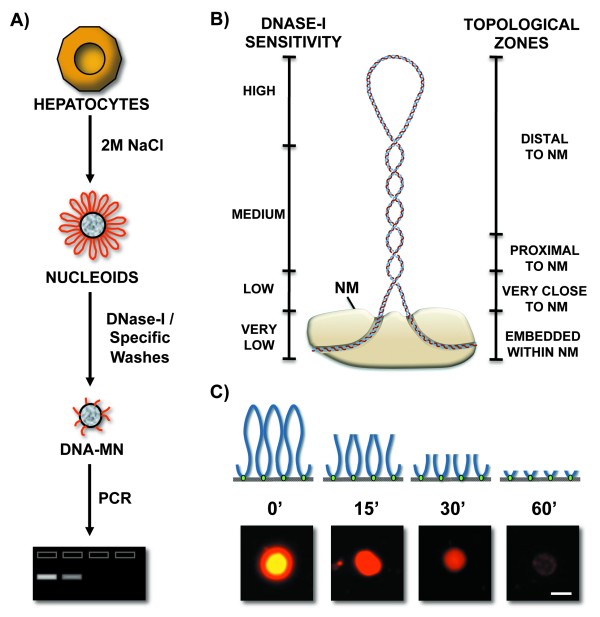
**Procedure for mapping the position of loop DNA sequences relative to the NM**. A) Nucleoids prepared from freshly isolated primary rat hepatocytes were incubated with DNase I so as to progressively digest the loop DNA. Nucleoid samples with partially digested NM-bound DNA, as shown in the drawing and fluorescent micrographs (C) were used for PCR amplification of target sequences. The amplicons were run in agarose gels and scored as present (+) or absent (-) by an image analysis software as a function of nucleoid-sample digestion time (C) and the corresponding topological zones relative to the NM defined by the kinetics of nucleoid-DNA digestion. B) Drawing illustrating the local topology along a typical supercoiled DNA loop that correlates with both sensitivity to DNase I and distance relative to the NM thus defining topological zones relative to the NM.

The high-salt resistant structural DNA loops plus the NM constitute a nucleoid (Figure 1A). Under the conditions of lysis employed to generate nucleoids the nuclear DNA remains essentially intact, although it lacks the nucleosome structure because of the dissociation of histones and most other nuclear proteins usually associated with DNA, yet the structural DNA loops remain topologically constrained by being anchored to the NM [24,25] thus being equivalent to closed DNA circles. Under such condition the DNA molecule undergoes structural stress resulting from two factors: the covalently linked backbones of the DNA strands are helicoidal but rigid while the low-energy hydrogen bonds between the stacked bases are quasi-statistical unions that continuously break apart and form again, such a situation poses the risk that the nucleotide bases may gyrate away from the double helix axis and become exposed. DNA naturally solves this structural- stress problem by further coiling upon its own axis thus avoiding exposure of the nucleotide bases, but becoming negatively supercoiled in a similar fashion as a pulled house-telephone cord [26,27]. Thus the naked DNA loops display a gradient of supercoiling that goes from lower to higher from tip to base of the loop, save for the fact that the structural properties of MARs are such that they also function as buffers or sinks of negative supercoiling [28,29] thus avoiding maximal supercoiling at the base of the loops (Figure 1B).

Indeed, nucleoids are also known as nuclear halos since exposure of such structures to DNA-intercalating agents like ethidium bromide leads to unwinding of the DNA loops that form a DNA halo around the NM periphery (Figure 1C). A typical structural DNA loop can be divided into four topological zones according to their relative proximity to the NM [23]. Each of these zones would manifest an identifiable behavior when exposed to non-specific nucleases that are sensitive to the local DNA topology such as DNase I (Figure 1B). We have previously shown that in nucleoid preparations the relative resistance of a given loop-DNA sequence to a limited concentration of DNase I is directly proportional to its proximity to the NM anchoring point [12,13], two main factors determine such property: (1) Steric hindrance resulting from the proteinaceous NM that acts as a physical barrier that relatively protects the naked loop DNA that is closer to the NM from endonuclease action. (2) The local degree of loop DNA supercoiling that is lower in the distal portions of the loop and higher in the regions proximal to the NM. Both factors only confer relative but not absolute DNase I-resistance to loop DNA. Supercoiling is a structural barrier against the action of non-specific endonucleases such as DNase I, that hydrolyze the DNA backbone by a single-strand cleavage (nicking) mechanism [30]. Theoretically a single nick in a DNA loop would lead to its complete unwinding but the loss of supercoiling will not occur instantaneously and so the actual rate of supercoiling loss in a large sample of nucleoids exposed to a limited concentration of DNase I reflects the gradient of supercoiling (Figure 1B) present along the average DNA loop [25,27]. Thus in a large sample of nucleoids exposed to a limited concentration of DNase I there is a consistent trend in which the loop-DNA sensitivity to the enzyme is inversely proportional to its distance relative to the NM (Figure 1C) and so on average the distal regions of the loop are digested first while the regions closer to the NM are digested later [12,13].

**Figure 2:**
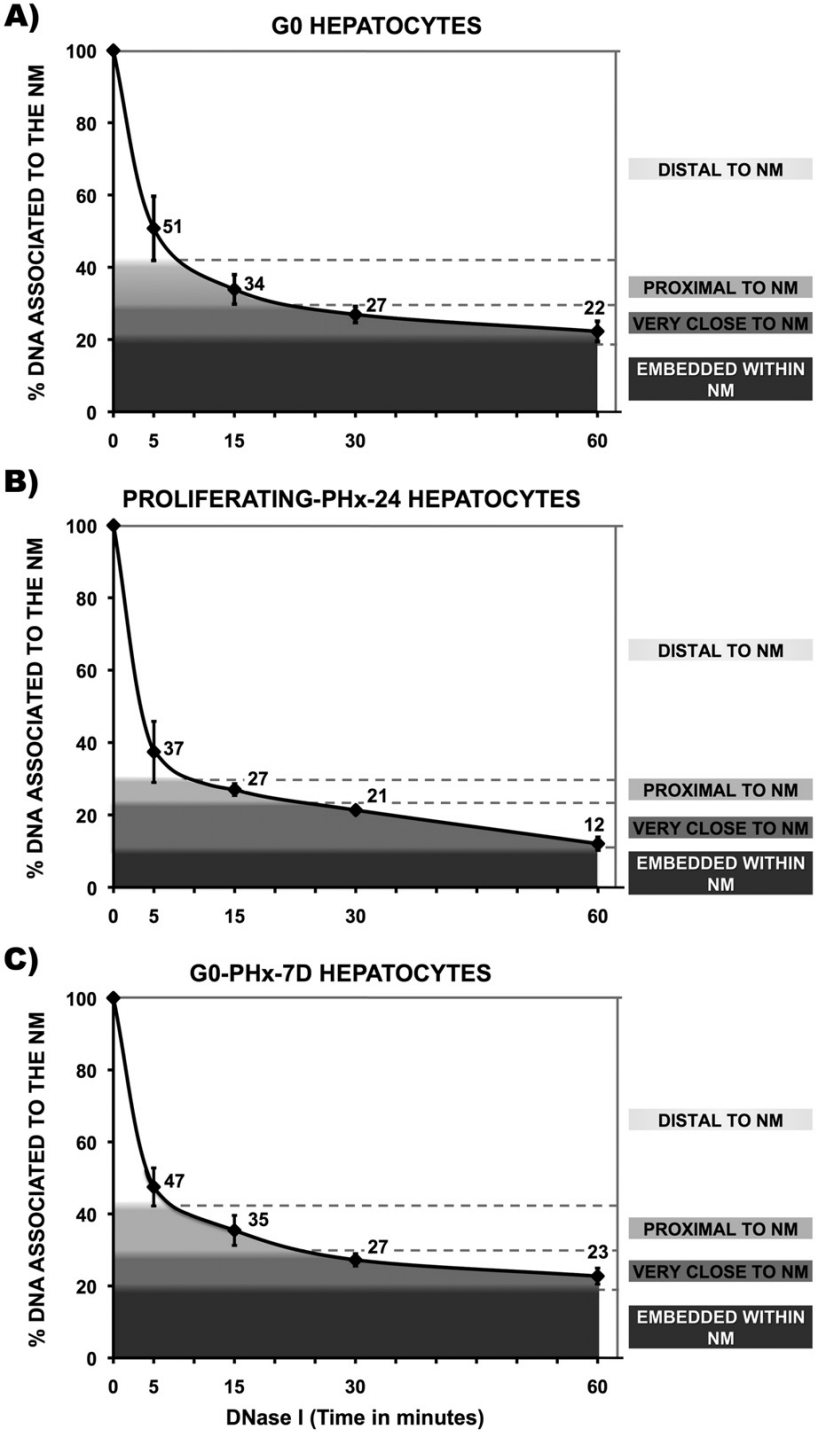
**Kinetics of nucleoid-DNA digestion**. Nucleoids from freshly isolated primary rat hepatocytes were digested with a limited concentration of DNAse I (0.5 U/ml) for different times. Each time-point value is the average of separate experiments with samples obtained from separate animals: control G0 hepatocytes (n = 9). Proliferating (PHx-24) hepatocytes 24 h after partial hepatectomy (n = 5). Restored G0 hepatocytes (PHx-7D) 7 days after partial hepatectomy (n = 5). Bars indicate the corresponding S.D. The topological zones relative to the NM (distal, proximal, very close and embedded within the NM) correspond to decreasing percentages of total DNA bound to the NM and were defined according to the corresponding decreasing slopes in each curve. Slopes for control G0 hepatocytes: 0 - 5 min = -9.80; 5 - 15 min = -1.70; 15 - 30 min = - 0.47; 30 - 60 min = -0.17. Slopes for proliferating PHx- 24 hepatocytes: 0 - 5 min = - 12.52; 5 - 15 min = - 1.04; 15 - 30 min = - 0.38; 30 - 60 min = - 0.31. Slopes for G0 PHx-7D hepatocytes: 0 - 5 min = - 10.50; 5 - 15 min = - 1.21; 15 - 30 min = - 0.55; 30 - 60 min = - 0.15.

Treatment of nucleoids from quiescent (G0), freshly isolated rat hepatocytes, with a limited concentration of DNase I (0.5 U/ml) produces a highly reproducible kinetics of digestion of loop DNA (Figure 2A). It is possible to identify in the digestion curve three different phases [23]: the first corresponds to a very fast kinetics of digestion that removes some 60% of the total DNA associated to the NM, within the first five minutes. Such a DNA corresponds to the loop fraction distal to the NM, in such fraction relatively minor DNA supercoiling is the main barrier to the endonuclease action (Figure 1B). Moreover, as the DNase I nicks the loop DNA, each nick becomes a point of further DNA unwinding, thus increasing the reduction of loop supercoiling in time and so making more accessible the loop DNA to the action of the endonuclease. The second phase that lasts about 10 minutes shows a reduced kinetics of digestion in which some further 10% loop DNA is removed. The third and slowest phase, lasting 45 minutes, shows the removal of some further 10% of total DNA associated with the NM. This very slow kinetics reflects the steric hindrance resulting from the proximity between the loop DNA and the NM proteins that act as physical barriers against the action of DNase I upon the loop DNA that is very close to the NM. Finally, there is about 20% of total DNA that remains bound to the NM even after 60 minutes of treatment with DNase I. This fraction corresponds to the DNA that is actually embedded within the NM and so it is rather inaccessible to the limited concentration of DNase I used. Such a DNA is easily identifiable in the digestion curves as the fraction that is basically resistant to digestion by limited DNase I so that the local slope becomes close to zero (Figure 2A) and remains like that even after very long incubation times. Indeed, it is known that in non-dividing cells the DNA embedded within the NM is very resistant to DNase I action and there is a fraction corresponding to some 2% of the total DNA that is basically non-digestible even when exposed to high concentrations of the enzyme. This fraction corresponds to fragments with average length of 1.6 kb in rat hepatocytes [16], likely to represent the regions that include the actual MARs (LARs) anchored to the NM.

The surgical ablation of approximately 70% of the rat liver mass leads to massive, synchronized re-entry into the cell cycle of more than 95% of the residual hepatocytes, this is followed by a well characterized first large peak of DNA synthesis at 24 h, followed by a smaller peak between 36 and 48 h [22,31]. Restoration of the original liver mass requires some 1.6 proliferative cycles per residual hepatocyte and then they gradually return to the G0 quiescent state. The whole regeneration process usually lasts 7 days after which there is full recovery of the liver function [32]. Nucleoids from primary rat hepatocytes isolated at 24 h after partial hepatectomy and treated with DNase I show a faster kinetics of loop DNA digestion (Figure 2B). The fact that the residual DNA attached to the NM after the first five minutes of digestion is just some 30% of the total DNA suggests that DNA loop supercoiling is somehow being reduced by the actual process of DNA synthesis making DNA more accessible to the endonuclease and so the residual DNA attached to the NM after 60 min of digestion is about half the value of the G0 samples (Figure 2B), indicating greater overall sensitivity of DNA to the endonuclease. This phenomenon has been previously described in nucleoids from cells undergoing DNA synthesis [33,34]. Yet, nucleoids from rat hepatocytes isolated 7 days after partial hepatectomy display a kinetics of DNA digestion that is quite similar to the original displayed by undisturbed G0 hepatocytes and so, the amount of endonuclease-resistant DNA embedded within the NM is basically the same (Figure 2A and 2C) indicating that the original local topology of the corresponding structural DNA loops has been fully restored in the newly-quiescent hepatocytes of the regenerated liver.

### Sequential movement of loop DNA relative to the NM correlates with DNA replication

**Figure 3:**
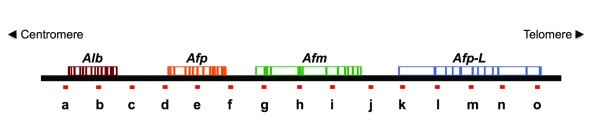
**Target sequences in the genomic region containing members of the rat albumin gene family**. The 162 kbp genomic region is located in chromosome 14 and includes the members of the rat albumin gene family: *Alb *(albumin), *Afp *(alpha-fetoprotein), *Afm *(afamin), *Afp-L *(pseudo-gene similar to *Afp*). The letters indicate the location of the target DNA sequences to be positionally mapped relative to the NM.

**Figure 4:**
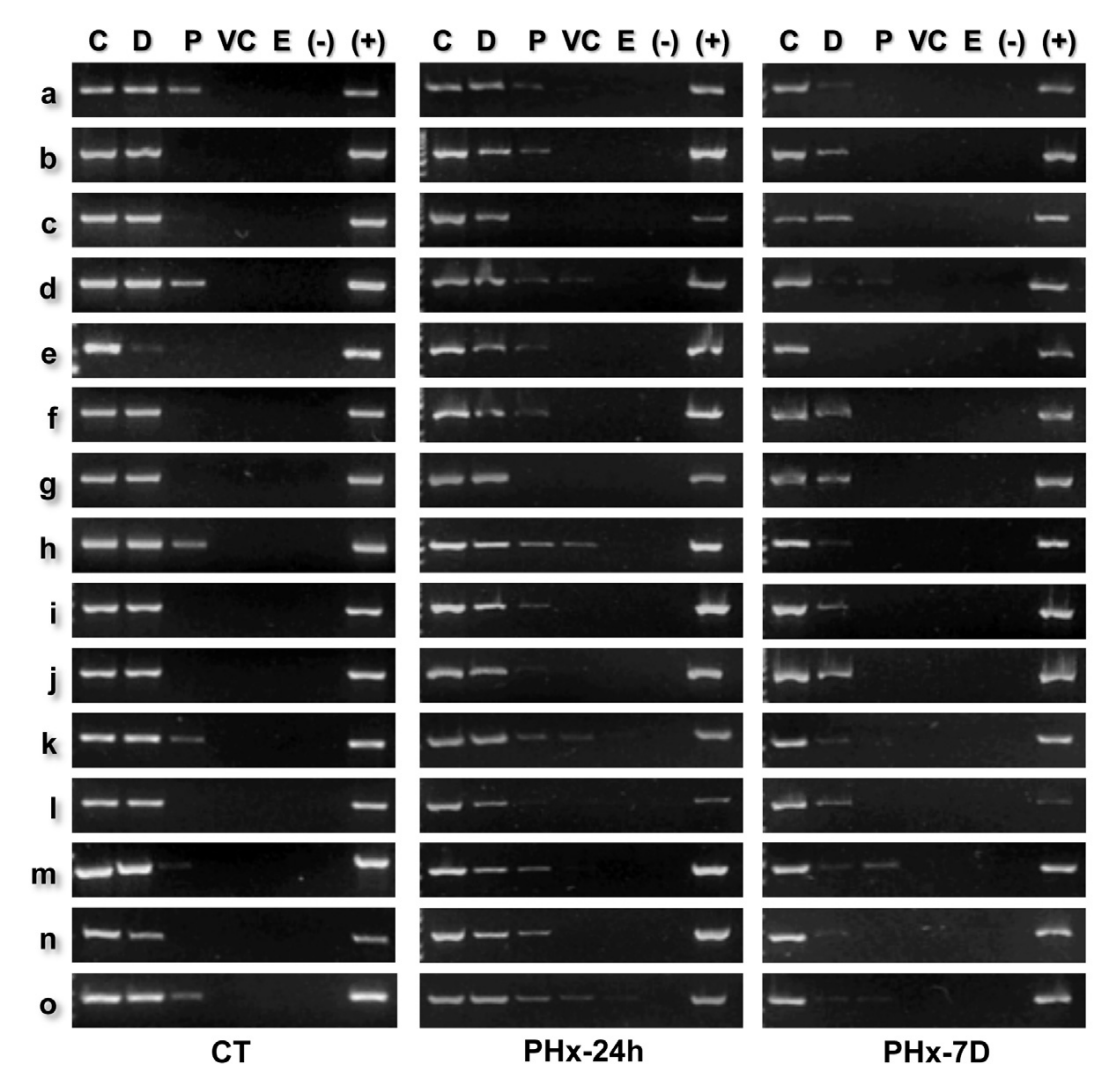
**Positional mapping relative to the NM of specific target sequences**. The position relative to the NM of specific target sequences along the 162 kbp rat albumin gene-family genomic region was determined by PCR. Nucleoids from rat hepatocytes were treated with DNase I (0.5 U/ml) for different times (Figure 2). The residual NM-bound DNA in the partially digested samples was directly used as template for PCR amplification of the chosen target sequences (a - o). The specific amplicons were resolved in 2% agarose gels and stained with ethidium bromide (0.5 μg/ml). C, 0' digestion-time control. The amplicons were scored either as positive or negative as a function of endonuclease digestion time and for each topological zone relative to the NM, depending on whether or not they were detected by a digital image-analysis system (Kodak 1D Image Analysis Software 3.5) using the default settings. Topological zones relative to the NM: D, distal; P, proximal; VC, very close; E, embedded within the NM. (-) Negative control (no template); (+) positive control (pure genomic DNA as template). CT, control G0 hepatocytes; PHx-24 h, hepatocytes 24 h after partial hepatectomy; PHx-7 D, hepatocytes 7 days after partial hepatectomy. The amplification patterns were consistently reproduced in separate experiments with samples from independent animals (n ≥ 3).

The properties described in the first paragraph of the previous section coupled to direct PCR amplification of specific target sequences on partially digested nucleoid preparations allow the mapping of the relative position to the NM of any specific loop-DNA sequence [12], according to the protocol depicted in Figure 1. Thus, templates corresponding to DNA sequences located closer to the NM will consistently being amplifiable in partially-digested nucleoid samples corresponding to longer times of DNase I treatment than templates corresponding to sequences located more distal relative to the NM. Using a slight variant of this method we were previously able to determine in primary rat hepatocytes the in vivo structural DNA loop organization in a 162 kbp region encompassing several genes of the albumin gene family [23]. The region comprises five structural DNA loops [23]. We designed a set of fifteen small amplicons (< 547 bp) rather evenly spaced about every 10 kb along the aforementioned region (Figure 3 and Table 1). Previous studies have shown that the average size of the nuclear DNA fragments liberated by non-specific nucleases in rat hepatocytes is 0.8 kb [16]. Thus the DNA sequences to be mapped are ≤ 550 bp in length (Table 1) and so likely to be cut as whole units by the endonuclease instead of being progressively eroded by partial digestions. Therefore in our mapping protocol we score the specific templates as either present (amplifiable) or absent (non-amplifiable) as a function of endonuclease-digestion time, without considering the intensity of the amplicon signals but just whether such signals are detected or not by an image-analysis program (Kodak 1D Image Analysis Software 3.5), using the default settings. We established these criteria because in our topological-mapping approach [12,23] it is the average relative position to the NM anchoring point and not the actual template length the critical parameter that determines the average sensitivity to DNase I of each sequence mapped. Thus absence of amplified product at a given digestion time-point indicates that the relative abundance of the target template has fallen to a non-amplifiable level within the large nucleoid population analyzed in each sample [10]. Using nucleoid samples from primary hepatocytes obtained from: control (G0), 24 h after partial hepatectomy (PHx-24) and 7 days after partial hepatectomy (PHx-7D) livers, we mapped the position relative to the NM of each of such fifteen amplicons. Figure 4 shows the typical amplification patterns of the target sequences in DNase I-treated nucleoid samples containing residual loop DNA corresponding to each of the four different topological zones relative to the NM (Figure 1B). The specific topological zones for each kind of partially-digested nucleoid hepatocyte sample were defined according to the specific kinetics of nucleoid DNA digestion (Figure 2A for G0 samples, Figure 2B for PHx-24, Figure 2C for PHx-7D).

**Table 1:** Sets of primers and corresponding amplicon sizes of target sequences

Amplicon	Forward Primer	Reverse Primer	Amplicon Length
**A**	TGGCAAACATACGCAAGGGA	GCGAAACACACCCCTGGAAA	275 pb
**B**	GAGGACAGTTAGTGCTGTAGGGTTG	CCTCCAACGAAGTTCCCAGAAT	547 pb
**C**	TCCTTTGTAACCAGGCAAGTGG	CCCATTTCCCAGATCCTTCACTCT	374 pb
**D**	CCCAGGGTCAGAGTATATCAGTGC	CGCTGAACGTATGTCTGAGTCA	305 pb
**E**	TGGTAGGCAGAGATGTGAGGAAAG	CCTGTTGTCCTAATGCTGGTCCTA	382 pb
**F**	CTGATCTTCAGGCAATATGGCAGG	TTGGCTGATGTCGTCTGGACA	393 pb
**G**	AAGGATAGGTGCTTGGCTGACA	GCCCTAACCCTGTGTGTGTATCTTG	504 pb
**h**	GATCACGTAACAACCCTGTCAGCT	CCTTCACAGCACCCGTCATACA	263 pb
**i**	GGTGCTGGGAATTTGACTAAGGC	TAAACTCAGGTGACAGGCTACGGC	465 pb
**j**	AGGAACCAGGGAATCGAGTGCT	AACTTGCGGGTGTTCTCTCCTT	392 pb
**k**	TGTCAGCATGATGGTGGCTCA	CTCGATTTGCCATGTCCTGTCT	252 pb
**l**	GGGCTGGGTCCATATTGCTTGA	ATGCTTTGGGCTTGCCTGAAG	373 pb
**m**	ACGACTTCCCTTCCTATCCACAG	GTAGAAAGTCGTTCTGGTTGCCAC	234 pb
**n**	CCCTAATCTTGCTGTGGTTTGG	TGAGAGCTGGGCAGACATCAA	355 pb
**O**	GGTGACAGTTGACAGAGAGCCTTC	GCTCCATGCTGACCTTGAAGTCTA	272 pb

Sets of primers and corresponding amplicon sizes defining fifteen target sequences along the 162 kbp genomic region of chromosome 14 containing members of the rat albumin gene family.

However, considering the topological zones defined by the kinetics of nucleoid DNA digestion in samples from undisturbed G0 hepatocytes as the original reference zones and using these zones for calibrating the actual positions relative to the NM of the target sequences in the three kinds of hepatocyte samples (G0, PHx-24 and PHx-7D) it is possible to establish a trend for all target sequences to become significantly closer to the NM at 24 h after partial hepatectomy when there is a peak of DNA synthesis (Table 2). On the other hand, in samples from 7 days after partial hepatectomy, when liver regeneration is basically complete, the target sequences consistently appear to have returned to their original positions relative to the NM (Table 2). Moreover, using the specific kinetics of nucleoid DNA digestion corresponding to G0, PHx-24 and PHx-7D (Figure 2) for locating each target sequence within a topological zone relative to the NM in each kind of nucleoid samples, it was confirmed a general trend for most target sequences to become closer to the NM at 24 after partial-hepatectomy, as well as the fact that all target sequences return to their basal positions at seven days after partial-hepatectomy (Table 3). Detailed analysis of data in Table 3 revealed the following: sequences **a **and **m **appear to remain immobile located in the zone proximal to the NM in both control and PHx-24 samples. Sequences **c **and **g **seem to remain immobile located in the zone distal to the NM in control, PHx-24 and PHx-7 samples. Eleven sequences (**b**, **d**, **e**, **f**, **h**, **I**, **j**, **k**, **l**, **n **and **o**) are all shifted in PHx-24 samples one further topological zone closer to the NM from their original position in control G0 samples, and then eight from such eleven sequences clearly return to their original position relative to the NM in PHx- 7D samples.

**Table 2:** Location of the target sequences within topological zones relative to the NM

	TOPOLOGICAL ZONES RELATIVE TO NM ACCORDING TO KINETICS OF DNA DIGESTION OF NUCLEOIDS FROM GO																
	
AMPLICON	HEPATOCYTES																
	
	CONTROL G0						PHX-24						PHX-7 D				
	
	C	D	P	VC	E		C	D	P	VC	E		C	D	P	VC	E
**a**	**+**	**+**	**+**	**-**	**-**		**+**	**+**	**+**	**+**	-*		**+**	**+**	-*	**-**	**-**
**b**	**+**	**+**	**-**	**-**	**-**		**+**	**+**	**+**	+*	**-**		**+**	**+**	**-**	**-**	**-**
**c**	**+**	**+**	**-**	**-**	**-**		**+**	**+**	**+**	-*	**-**		**+**	**+**	**-**	**-**	**-**
**d**	**+**	**+**	**+**	**-**	**-**		**+**	**+**	**+**	**+**	**-**		**+**	**+**	+*	**-**	**-**
**e**	**+**	**+**	**-**	**-**	**-**		**+**	**+**	**+**	+*	**-**		**+**	-*	**-**	**-**	**-**
**f**	**+**	**+**	**-**	**-**	**-**		**+**	**+**	**+**	**+**	**-**		**+**	**+**	**-**	**-**	**-**
**g**	**+**	**+**	**-**	**-**	**-**		**+**	**+**	**+**	-*	**-**		**+**	**+**	-*	**-**	**-**
**h**	**+**	**+**	**+**	**-**	**-**		**+**	**+**	**+**	**+**	**-**		**+**	**+**	-*	**-**	**-**
**i**	**+**	**+**	**-**	**-**	**-**		**+**	**+**	**+**	**+**	**-**		**+**	**+**	**-**	**-**	**-**
**j**	**+**	**+**	**-**	**-**	**-**		**+**	**+**	**+**	**+**	**-**		**+**	**+**	-*	**-**	**-**
**k**	**+**	**+**	**+**	**-**	**-**		**+**	**+**	**+**	**+**	-*		**+**	**+**	-*	**-**	**-**
**l**	**+**	**+**	**-**	**-**	**-**		**+**	**+**	**+**	+*	**-**		**+**	**+**	**-**	**-**	**-**
**m**	**+**	**+**	**+**	**-**	**-**		**+**	**+**	**+**	+*	**-**		**+**	**+**	+*	**-**	**-**
**n**	**+**	**+**	**-**	**-**	**-**		**+**	**+**	**+**	+*	**-**		**+**	**+**	**-**	**-**	**-**
**o**	**+**	**+**	**+**	**-**	**-**		**+**	**+**	**+**	**+**	-*		**+**	**+**	+*	**-**	**-**

Location of the fifteen target sequences from the 162 kbp genomic region studied within the topological zones relative to the NM. Such topological zones were defined according to the kinetics of nucleoid-DNA digestion in samples from control G0 hepatocytes obtained from normal rat liver (Figure 2A). The amplicons were scored either as positive or negative as a function of endonuclease digestion time and for each topological zone relative to the NM, depending on whether or not they were detected by a digital image-analysis system (Kodak 1D Image Analysis Software 3.5) using the default settings. G0, samples from normal rat liver hepatocytes. PHx-24, samples obtained from hepatocytes 24 h after partial hepatectomy. PHx-7 D, samples obtained from hepatocytes 7 days after partial hepatectomy. C, 0' digestion-time control. D, distal to the NM. P, proximal to the NM. VC, very close to the NM. E, embedded within the NM. All target sequences were mapped in repeated experiments with samples from separate animals (n ≥3). *Indicates that one of the repetitions gave a different result: -* in one experiment was positive; +* in one experiment was negative.

**Table 3:** Location of the target sequences relative to the NM according to the sample-specific kinetics

TOPOLOGICAL ZONES RELATIVE TO NM																	
	
AMPLICON	CONTROL G0						PHX-24						PHX-7D				
	
	C	D	P	VC	E		C	D	P	VC	E		C	D	P	VC	E
**a**	**+**	**+**	**+**	**-**	**-**		**+**	**+**	**+**	-*	-*		**+**	**+**	-*	**-**	**-**
**b**	**+**	**+**	**-**	**-**	**-**		**+**	**+**	+*	**-**	**-**		**+**	**+**	**-**	**-**	**-**
**c**	**+**	**+**	**-**	**-**	**-**		**+**	**+**	-*	**-**	**-**		**+**	**+**	**-**	**-**	**-**
**d**	**+**	**+**	**+**	**-**	**-**		**+**	**+**	**+**	+*	**-**		**+**	**+**	+*	**-**	**-**
**e**	**+**	**+**	**-**	**-**	**-**		**+**	**+**	+*	**-**	**-**		**+**	-*	**-**	**-**	**-**
**f**	**+**	**+**	**-**	**-**	**-**		**+**	**+**	**+**	-*	**-**		**+**	**+**	**-**	**-**	**-**
**g**	**+**	**+**	**-**	**-**	**-**		**+**	**+**	-*	**-**	**-**		**+**	**+**	-*	**-**	**-**
**h**	**+**	**+**	**+**	**-**	**-**		**+**	**+**	**+**	**+**	**-**		**+**	**+**	-*	**-**	**-**
**i**	**+**	**+**	**-**	**-**	**-**		**+**	**+**	**+**	**-**	**-**		**+**	**+**	**-**	**-**	**-**
**j**	**+**	**+**	**-**	**-**	**-**		**+**	**+**	**+**	**-**	**-**		**+**	**+**	-*	**-**	**-**
**k**	**+**	**+**	**+**	**-**	**-**		**+**	**+**	**+**	**+**	-*		**+**	**+**	-*	**-**	**-**
**l**	**+**	**+**	**-**	**-**	**-**		**+**	**+**	+*	**-**	**-**		**+**	**+**	**-**	**-**	**-**
**m**	**+**	**+**	**+**	**-**	**-**		**+**	**+**	**+**	-*	**-**		**+**	**+**	+*	**-**	**-**
**n**	**+**	**+**	**-**	**-**	**-**		**+**	**+**	+*	**-**	**-**		**+**	**+**	**-**	**-**	**-**
**o**	**+**	**+**	**+**	**-**	**-**		**+**	**+**	**+**	**+**	-*		**+**	**+**	+*	**-**	**-**

Location of the fifteen target sequences from the 162 kbp genomic region studied within the specific topological zones relative to the NM. Such topological zones were defined according to the specific kinetics of nucleoid-DNA digestion for each kind of nucleoid sample (Figure 2A for G0; Figure 2B for PHx-24; Figure 2C for PHx-7D samples). The amplicons were scored either as positive or negative as a function of endonuclease digestion time and for each topological zone relative to the NM, depending on whether or not they were detected by a digital image-analysis system (Kodak 1D Image Analysis Software 3.5) using the default settings. G0, samples from normal rat liver hepatocytes. PHx-24, samples obtained from hepatocytes 24 h after partial hepatectomy. PHx-7 D, samples obtained from hepatocytes 7 days after partial hepatectomy. C, 0' digestion-time control. D, distal to the NM. P, proximal to the NM. VC, very close to the NM. E, embedded within the NM. All target sequences were mapped in repeated experiments with samples from separate animals (n ≥3). *Indicates that one of the repetitions gave a different result: -* in one experiment was positive; +* in one experiment was negative.

**Figure 5:**
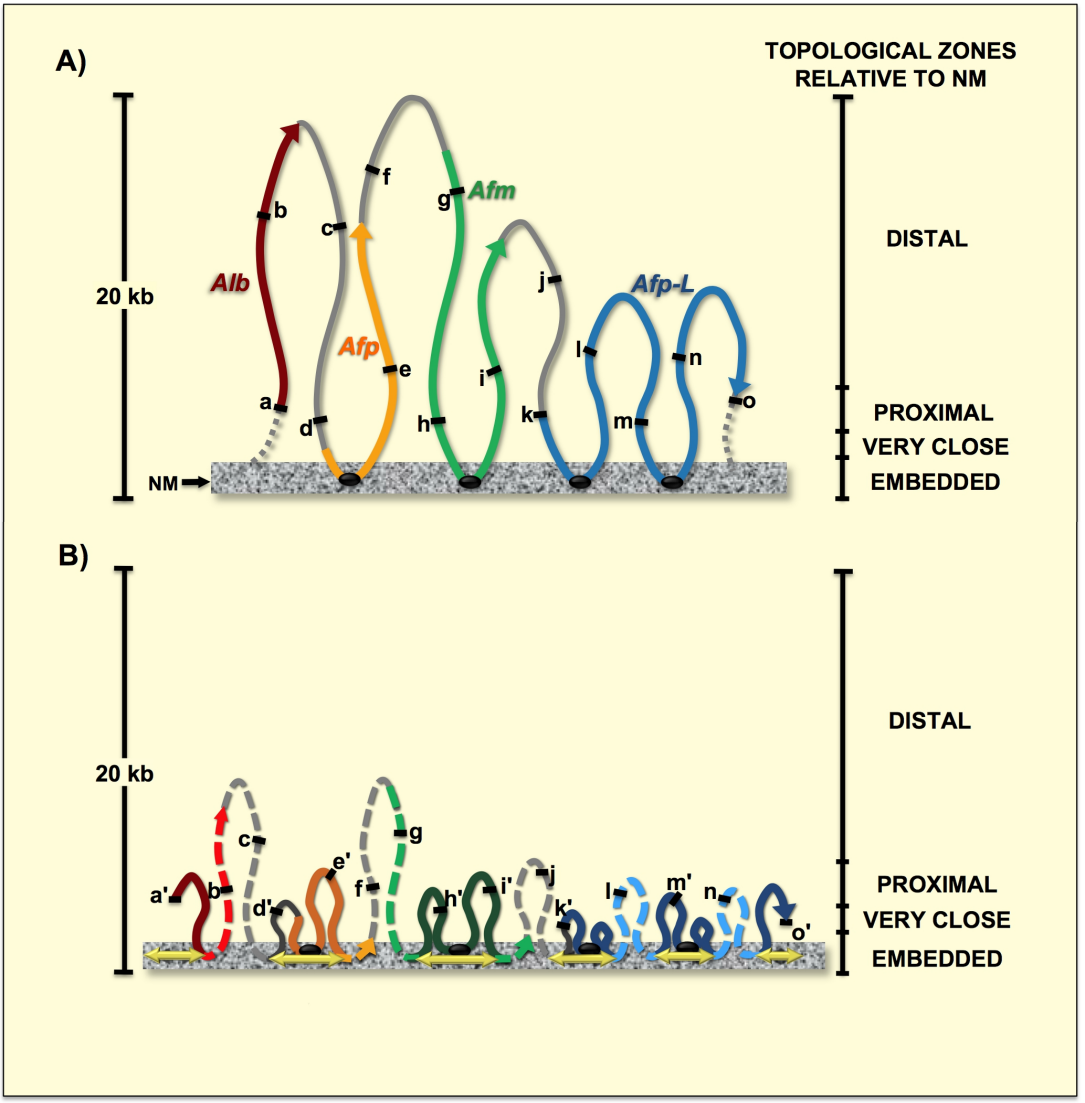
**Changes in the configuration of the structural DNA loops as a function of DNA replication**. A) Structural DNA loop organization of the 162 kbp region containing the rat albumin locus in undisturbed, control G0 hepatocytes. The letters (a-o) indicate the position of the corresponding target sequences relative to the NM. Color lines indicate the position of the corresponding named gene. The small dashed lines indicate projected loop regions outside of the region studied. The black ovals within the NM correspond to putative LARs. B) Average configuration of the structural DNA loops in the same region at the peak of DNA synthesis (24 h after partial hepatectomy) deduced from the results in Tables 2 and 3. Dashed lines indicate the segment of the corresponding gene possibly awaiting replication. Bold-color lines indicate the daughter duplexes containing one parental strand and one newly made strand, that are extruded in loops as the parental duplex slides through the fixed replication sites on the NM (parental loops shrink whereas daughter loops grow). Letters (b...) indicate target sequences to be replicated. Letters with apostrophe (a'...) indicate target sequences that have been replicated already. The double-headed arrows in the NM correspond to active replication forks derived from putative origins of replication located close to the LARs. In this representation the early-replicating LARs (black ovals) become attached to the NM immediately after replication, thus producing tiny but transient duplicate daughter loops that should disappear once the whole region has been duplicated.

Since the target sequences correspond to sites located at different points along the five structural loops comprising the region studied (Figure 5A), these results suggest that at 24 h after partial hepatectomy the DNA loops are somehow reeled or pulled progressively towards the NM where the replication factories are assembled (Figure 5B), recovering later on their original configuration. This is confirmed by the fact that the positions of most target sequences relative to the NM in rat hepatocytes obtained 7 days after partial hepatectomy (when liver regeneration is complete) are basically the same as those in undisturbed G0 controls (Table 2 and 3).

**Figure 6:**
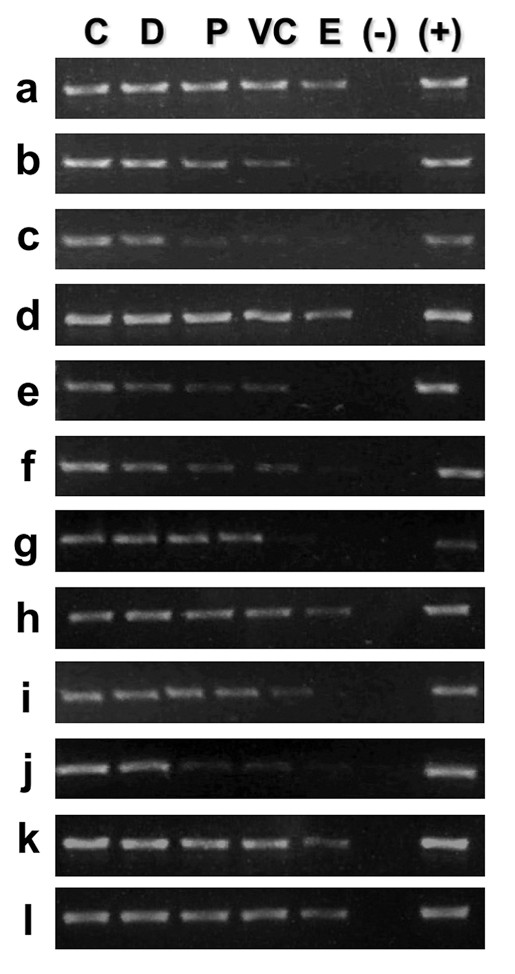
**Positional mapping relative to the NM using a higher DNA template concentration**. Positional mapping relative to the NM of specific target sequences along the 162 kbp rat albumin gene-family genomic region by PCR using a higher DNA template concentration. Nucleoids from G0 rat hepatocytes were treated with DNase I (0.5 U/ml) for different times (Figure 2A). The residual NM-bound DNA in the partially digested samples was directly used as template for PCR amplification of the chosen target sequences (a - o). For these experiments the nuclear matrix-bound DNA template was increased six-fold (from 10 to 60 ng) and the DNA polymerase concentration was doubled (from 0.7 to 1.25 U) in order to facilitate the amplification of target sequences embedded within the NM. The specific amplicons were resolved in 2% agarose gels and stained with ethidium bromide (0.5 μg/ml). C, 0' digestion-time control. The amplicons were scored either as positive or negative as a function of endonuclease digestion time and for each topological zone relative to the NM, depending on whether or not they were detected by a digital image-analysis system (Kodak 1D Image Analysis Software 3.5) using the default settings. Topological zones relative to the NM: D, distal; P, proximal; VC, very close; E, embedded within the NM. (-) Negative control (no template); (+) positive control (pure genomic DNA as template). For detailed analysis of results see Table 4.

Considering that the topology-dependent resistance to DNase I of target sequences is relative but not absolute, the extinction of a target amplicon at a given DNA-digestion time corresponds to the actual reduction of the target template to a non-amplifiable concentration within the nucleoid population analyzed. Moreover, DNA embedded within the NM is poorly accessible to externally added polymerases and so it is non- amplifiable under our standard experimental conditions. Nevertheless, a six-fold rise in the concentration of NM-bound DNA template (from 10 to 60 ng) allows detection of some target sequences in the NM-embedded fraction even in control G0 samples (Figure 6) but this situation leads to a general shift of all target sequences towards closer positions relative to the NM. Moreover, the location of some target sequences drifts between adjacent topological zones from one experiment to another due to the artifactual rise in the relative abundance of the target-sequence templates (Table 4). Since our results correspond to an average snapshot of the S phase in vivo the loop DNA configuration depicted (Figure 5B) is very likely the sum of different hepatocyte subpopulations at different stages within the S phase. Therefore, under our standard experimental conditions only those nucleoids subpopulations in which the target sequence is not actually embedded within the NM may contain the corresponding amplifiable template. Moreover, LARs themselves cannot be reliable targets for PCR amplification on NM- bound templates since they usually contain repetitive sequences that preclude the design of highly-specific and efficient primers [23] but they are also poorly accessible for amplification by being embedded within the NM.

**Table 4:** Location of the target sequences relative to the NM using a higher DNA template concentration

AMPLICON	TOPOLOGICAL ZONES RELATIVE TO NM					
	
	C	D	P	VC	E	n
**a**	+	+	+	+	+	**4**
**b**	+	+	+	+	+/-	**2**
**c**	+	+	+	+	+/-	**2**
**d**	+	+	+	+	+	**4**
**e**	+	+	+	+	+/-	**2**
**f**	+	+	+	+	-	**2**
**g**	+	+	+	+/-	-	**2**
**h**	+	+	+	+	+	**4**
**i**	+	+	+	+	+	**2**
**j**	+	+	+	+	+/-	**2**
**k**	+	+	+	+	+	**3**
**l**	+	+	+	+	+/-	**3**

Location in control G0 hepatocytes of the fifteen target sequences from the 162 kbp genomic region studied within the topological zones relative to the NM using a higher DNA template concentration. Such topological zones were defined according to the kinetics of nucleoid-DNA digestion in samples from control G0 hepatocytes obtained from normal rat liver (Figure 2A). For these experiments the nuclear matrix-bound DNA template was increased six-fold (from 10 to 60 ng) and the DNA polymerase concentration was doubled (from 0.7 to 1.25 U) in order to facilitate the amplification of target sequences embedded within the NM. The amplicons were scored either as positive or negative as a function of endonuclease digestion time and for each topological zone relative to the NM, depending on whether or not they were detected by a digital image-analysis system (Kodak 1D Image Analysis Software 3.5) using the default settings. C, 0' digestion-time control. D, distal to the NM. P, proximal to the NM. VC, very close to the NM. E, embedded within the NM. All target sequences were mapped in repeated experiments with samples from separate animals (n indicated in the table). +/- indicates that one experiment was positive and the other experiment was negative.

### Movement of unrelated DNA sequences towards the NM correlates with the replicating status of the cells in vivo

**Figure 7:**
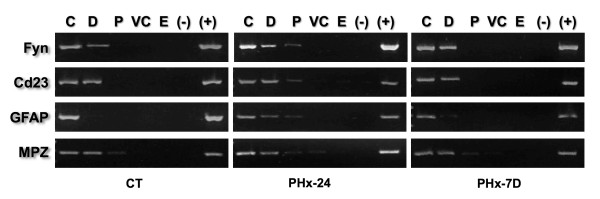
**Positional mapping relative to the NM of unrelated gene sequences**. Nucleoids from rat hepatocytes were treated with DNase I (0.5 U/ml) for different times. The residual NM-bound DNA in the partially digested samples was directly used as template for PCR amplification of small target sequences (Table 2) located in the 5' ends of the corresponding genes (*Fyn, CD23, GFAP *and *MPZ*). Each of such genes is located in a different chromosome and thus they represent separate chromosome territories within the nucleus. The specific amplicons were resolved in 2% agarose gels and stained with ethidium bromide (0.5 μg/ml). CT, control G0 hepatocytes; PHx-24 h, hepatocytes 24 h after partial hepatectomy; PHx-7 D, hepatocytes 7 days after partial hepatectomy C, 0' digestion-time control. Topological zones relative to the NM: D, distal; P, proximal; VC, very close; E, embedded within the NM. (-) Negative control (no template); (+) positive control (pure genomic DNA as template). The amplification patterns were consistently reproduced in separate experiments with samples from independent animals (n ≥ 3).

The fifteen sequences analyzed so far belong to a genomic region that comprises three genes and one pseudo-gene of the albumin gene family (Figure 3) and are located within five consecutive structural DNA loops corresponding to 162 kb of chromosome 14 in the rat (Figure 5A). Thus, we also mapped the position relative to the NM of four target- sequences corresponding to four unrelated genes each located in a different rat chromosome (Table 5) in order to corroborate whether nuclear DNA moves towards the NM during DNA replication. Thus amplicons belonging to the 5' ends of the genes *GFAP, CD23 *(also known as *Feer2*), *MPZ *and *Fyn *were positionally mapped relative to the NM in G0, PHx-24 and PHx-7D samples. The results indicate that all target sequences shift from their original locations to a topological zone closer to the NM at 24 h after partial-hepatectomy when the peak of DNA synthesis occurs, and then recover their original positions relative to the NM as shown in PHx-7D samples obtained when the liver regeneration is complete (Figure 7 and Table 6). This suggests that movement of DNA towards the NM is a general nuclear phenomenon during DNA synthesis in vivo and supports the notion that during replication it is the DNA template that moves towards the replication factories on the NM.

**Table 5:** Target sequences corresponding to four unrelated genes from separate chromosomes

Amplicon	Chromosome	Forward Primer	Reverse Primer	Amplicon Length
**GFAP**	10	TCCAGCCCGTCCCTCAATAA	TCCCGAAGTTCTGCCTGGTAA	418 bp
**CD23**	12	TAGGAGACGATGCTGCTGTGCA	CGTGGGAAGAGGATCAGACAAGAA	284 bp
**MPZ**	13	CTTGCCCCTACCCCAGCTAT	TCTCCTTGGCTGGCTCTCAAT	184 bp
**Fyn**	20	ACACAATGCTGATCTAGTCGTGGC	CACATCTGTGTTCATCACTGTCCG	340 bp

Sets of primers and corresponding amplicon sizes defining target sequences in four unrelated genes located in different rat chromosomes. All the amplicons were designed for the 5' end of the corresponding gene.

**Table 6:** Location of the target sequences of unrelated genes relative to the NM

	TOPOLOGICAL ZONES RELATIVE TO NM ACCORDING TO KINETICS OF DNA DIGESTION OF NUCLEOIDS FROM GO HEPATOCVTES																
	
Amplicon	CONTROL G0						PHX-24						PHX-7D				
	
	C	D	P	VC	E		C	D	P	VC	E		C	D	P	VC	E
**Fyn**	**+**	**+**	**-**	**-**	**-**		+	+	+	+	-		+	+	-	-	-
**CD23**	**+**	**+**	**-**	**-**	**-**		+	+	+	+	-		+	+	-	-	-
**GFAP**	**+**	**+**	**-**	**-**	**-**		+	+	+	+	-		+	+*	-	-	-
**MPZ**	**+**	**+**	**+**	**-**	**-**		+	+	+	+	-*		+	+	+*	-	-

	**TOPOLOGICAL ZONES RELATIVE TO NM**																
	
**Amplicon**	**CONTROL GO**						**PHX-24**						**PHX-7D**				
	
	**C**	**D**	**P**	**VC**	**E**		**C**	**D**	**P**	**VC**	**E**		**C**	**D**	**P**	**VC**	**E**

**Fyn**	**+**	**+**	**-**	**-**	**-**		**+**	**+**	**+**	**-**	**-**		**+**	**+**	**-**	**-**	**-**
**CD23**	**+**	**+**	**-**	**-**	**-**		**+**	**+**	**+**	-*	**-**		**+**	**+**	**-**	**-**	**-**
**GFAP**	**+**	**+**	**-**	**-**	**-**		**+**	**+**	**+**	**-**	**-**		**+**	+*	**-**	**-**	**-**
**MPZ**	**+**	**+**	**+**	**-**	**-**		**+**	**+**	**+**	**+**	-*		**+**	**+**	+*	**-**	**-**

Location of four target sequences each belonging to a different gene in a separate chromosome, within the topological zones relative to the NM. In the upper section of the table the topological zones were defined according to the kinetics of nucleoid-DNA digestion in samples from control G0 hepatocytes obtained from normal rat liver (Figure 2A). In the lower section of the table the topological zones were defined according to the specific kinetics of nucleoid-DNA digestion for each kind of nucleoid sample (Figure 2A for G0; Figure 2B for PHx-24; Figure 2C for PHx-7D samples). The amplicons were scored either as positive or negative as a function of endonuclease digestion time and for each topological zone relative to the NM, depending on whether or not they were detected by a digital image-analysis system (Kodak 1D Image Analysis Software 3.5) using the default settings. G0, samples from normal rat liver hepatocytes. PHx-24, samples obtained from hepatocytes 24 h after partial hepatectomy. PHx-7 D, samples obtained from hepatocytes 7 days after partial hepatectomy. C, 0' digestion-time control. D, distal to the NM. P, proximal to the NM. VC, very close to the NM. E, embedded within the NM. All target sequences were mapped in repeated experiments with samples from separate animals (n ≥3). *Indicates that one of the repetitions gave a different result: -* in one experiment was positive; +* in one experiment was negative.

## Discussion

There is important evidence that DNA replication in metazoan cells occurs in macromolecular complexes or factories assembled at rather fixed sites upon the NM [15,20,21]. DNA constitutes a long, thin but helical template and this poses important restrictions to the regular movement of tracking polymerases along such a template as well as it creates topological dead-ends resulting from the fact that in vivo, DNA is naturally underwound and negatively supercoiled so as to dissipate structural stress [26,27]. Tracking of polymerases along DNA generates torsional stress that may be relived by the action of topoisomerases; however, this situation poses the need for a highly-synchronized action of several independent molecular factors otherwise DNA replication may become stalled very often during the S phase. Moreover, considering the enormous size of the actual replication factories that dwarf the DNA template it would be more reasonable from an energy-wise perspective if the template moves towards the replication factories instead of the opposite and this would also reduce the possibility of structural, topological conundrums resulting from DNA replication [20]. Nascent DNA has been found consistently associated with the NM [16,35-37], and well-characterized origins of replication are dynamically bound to the NM in late G1 before the start of the S phase [38]. We have previously shown that in primary hepatocytes synchronized in vivo for DNA replication several genes located in separate chromosomes move towards the NM during the S phase [10]. These facts support the notion that DNA replicates in vivo at sites located on the NM.

Cells in culture, even primary cells, are prone to a number of artifacts that may affect the process of DNA replication, such as the fact that the elastic modulus of the cell culture recipient or the viscosity of the cell culture substrate may dramatically alter the rate of DNA replication [39,40], also the procedures for synchronizing cells in the S phase in vitro lead to various artifacts [41]. However, the present results were obtained with samples from freshly isolated rat hepatocytes that were shifted from G0 to S phase in a highly synchronous fashion by the partial hepatectomy. The comparative kinetics of nucleoid DNA digestion with DNase I between G0 and proliferating rat hepatocytes (Figure 2) is consistent with previous studies showing that replicating DNA bound to the NM is very sensitive to DNase I, thus supporting the notion that DNA synthesis occurs at sites located very close to the NM [16]. Indeed, the overall faster kinetics of nucleoid DNA digestion observed in the samples from PHx-24 hepatocytes may result from the action of DNA helicases necessary for DNA replication that speed-up the loss of loop DNA supercoiling which is a topological barrier to DNase I action. Moreover, the amount of DNA embedded within the NM that is resistant to the endonuclease is also reduced by almost 50% in hepatocytes that are synthesizing DNA (Figure 2A and 2B), this phenomenon can be accounted for by the fact that replication forks, in the replication factories organized upon the NM, contain single-stranded DNA [42] that is easily cleaved by DNase I and so the actual fraction of double-stranded DNA embedded within the NM (highly-resistant to DNase I) is reduced during the S phase in vivo. This conclusion is supported by the observation that in nucleoid samples from hepatocytes that have returned to quiescence as the liver regeneration has been completed, the amount of DNA embedded within the NM and highly-resistant to DNase I is basically the same as that in the undisturbed quiescent hepatocytes (Figure 2A and 2C), indicating that the absence of active replication forks reduces significantly the sensitivity of the DNA embedded within the NM to DNase I. Moreover, in PHx-7D samples the overall kinetics of nucleoid DNA digestion is basically the same as that in undisturbed G0 controls (Figure 2A and 2C) suggesting that loop-DNA supercoiling has been fully recovered in such newly-quiescent hepatocytes.

Applying elementary topological principles such as that points in a deformable string (DNA) can be positionally mapped relative to a position-reference invariant (NM), and that from such a mapping it can be deduced the configuration of the string in third dimension [43], the topological configuration of the DNA loops during the S phase can be directly deduced from the corresponding positions of the target sequences relative to the NM (Table 3). However, it is important to stress that we are actually monitoring the average DNA loop arrangement in a large number of hepatocytes and so our mapping approach must be regarded as topological (non-metric). Our PHx-24 samples correspond to an average snapshot of the S phase in a large number of hepatocyte nucleoids and so the most likely average configuration at the peak of DNA synthesis of the structural loops studied is depicted in Figure 5B. Indeed, such an average snapshot of the S phase is possible because of the relatively high synchronicity of DNA synthesis at 24 h post-hepatectomy [22,31,32] since at earlier and latter hours around the main peak of DNA synthesis the location of target sequences relative to the NM becomes erratic (data not shown). Yet, under our standard experimental conditions the positional mappings of target sequences relative to the NM at 24 h post-hepatectomy are highly reproducible among experiments (Figure 4). It must be stressed that the highly reproducible and widely documented main peak of DNA synthesis observed at 24 h post-partial hepatectomy in young-adult laboratory rats [22,31,32] is the composite average signal of millions of hepatocytes of the regenerating liver that might be individually found in different stages of the S phase (early, middle or late) or even beyond or out from the S phase. In the same way, the data on the topological positional mapping of target sequences relative to the NM in our PHx-24 samples (Table 2 and 3) represent the most commonly detectable pattern of amplification signals of the corresponding target sequences (Figure 4). Such a pattern of amplification signals becomes quite reproducible at 24 h post-partial hepatectomy suggesting a direct correlation with the well known fact that at that time most hepatocytes in the regenerating liver are somewhere in the S phase, but it would be unwarranted to conclude anything about the detailed status of the replication factories, since all detailed studies on replication factories assembly and dynamics have been carried out in samples obtained from cells in culture and so far there are no established protocols for labeling such factories in whole animals undergoing liver regeneration. However, it has been shown that several components of the replication factories are organized upon the NM [18,21].

A detailed analysis of the positional changes displayed during the S phase by the target sequences located along the five DNA loops of the albumin gene family locus shows that two target sequences (**a**, **m**) apparently remain fixed in a position proximal to the NM, whereas another two target sequences (**c**, **g**) apparently remain fixed in a position distal to the NM and eleven target sequences (**b**, **d**, **e**, **f**, **g**, **h**, **i**, **j**, **k**, **l**, **n**, **o**) display a shift from their corresponding original positions towards the next closer topological zone relative to the NM (Table 3). Since our PHx-24 samples correspond to an average snapshot of the S phase in a large number of hepatocyte nucleoids, the possible average configuration at the peak of DNA synthesis of the DNA loops studied is that depicted in Figure 5B.

Therefore, assuming that DNA replication takes place in factories organized upon the NM we can interpret the resulting DNA loop arrangement in Figure 5B as follows: the **a **and **m **target sequences are among the first to be replicated, given their original proximity to the NM and to a relatively early-firing origin of replication, thus they may have already being replicated in most nucleoids and so daughter duplexes containing one parental strand and one newly made strand (that include the copied **a **and **m **sequences) have already being extruded from the replication complexes allowing such **a **and **m **sequences to regain their original positions relative to the NM. This situation will be detected in our experimental system as lack of any change in the already proximal position of the **a **and **m **sequences relative to the NM. However, the **c **and **g **sequences remain distal to the NM because they are located relatively far away from the active replication forks and so they have not been replicated yet in most nucleoids. This situation will be detected in our experimental system as lack of any change in the distal position relative to the NM of sequences **c **and **g**. All other target sequences display positions relative to the NM consistent to their relative proximity to putative replication forks on the NM, thus some sequences may have been already replicated (**d**, **e**, **h**, **i**, **k**, **o**) while others are about to be replicated when they finally reach the corresponding active replication fork (**b**, **f**, **j**, **l**, **n**).

**Figure 8:**
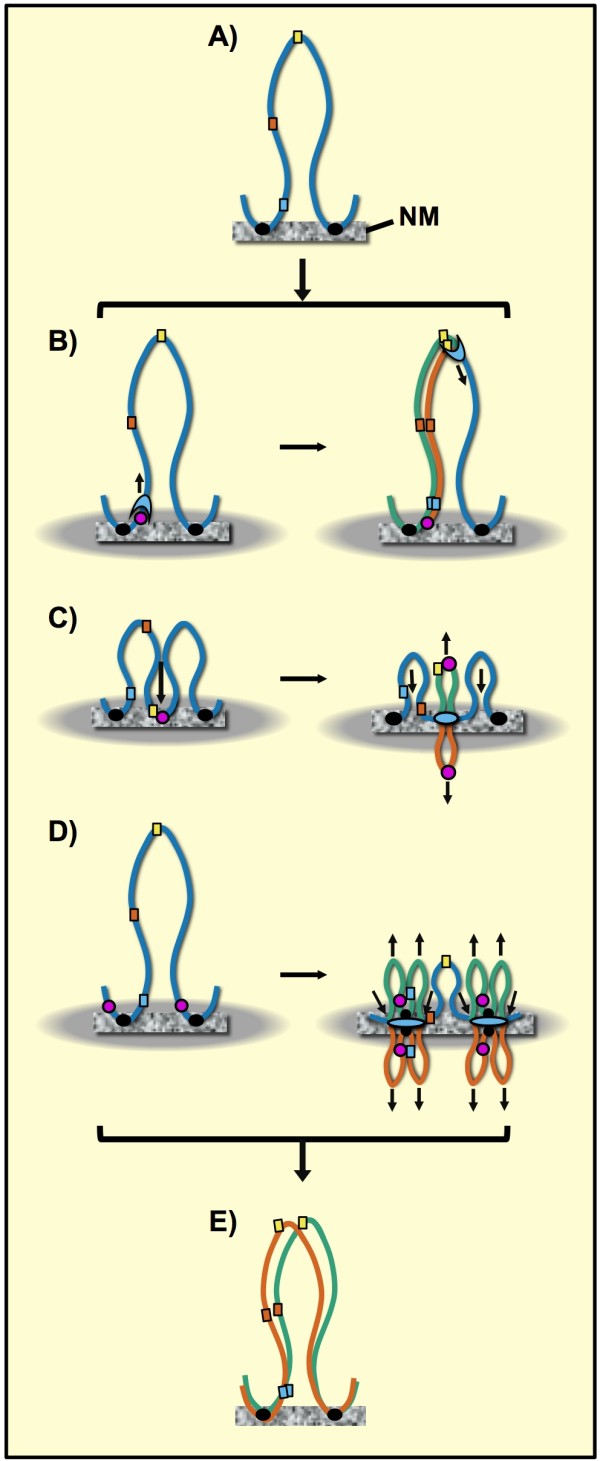
**Possible mechanisms for replicating DNA loops in vivo**. A) DNA loops are attached to the nuclear matrix (NM) by LARs sequences (black circles), the colored squares represent different sequences located along the loop DNA. B) Tracking model of DNA replication in which the replication complex (light blue) binds to a replication origin (purple circle) and moves along the template (blue string) while synthesizing the nascent daughter duplexes (orange and green strings). C) Reel-in model for DNA replication in which a replication origin (purple circle) originally at the tip of the DNA loop binds to the NM in late G1 reducing the actual loop size in half. The large grey oval represents the replication focus or factory. During the S phase the template (blue string) is pulled towards the replication complex on the NM that extrudes the newly replicated daughter duplexes (orange and green strings) that included the early-replicated origins. The LAR sequences (black circles) will be the last to be replicated. D) Reel-in model for DNA replication in which the origins are close to the LARs which are among the first loop DNA sequences to be duplicated. Replication begins close to the LARs but the newly-replicated LARs immediately attach to the NM as well as the newly-replicated origins that after initially firing gave origin to the actual replication forks (light blue ovals). Thus the growing daughter duplexes extruded from the replication complex on the NM are doubled in number, resulting in tiny but transient DNA loops that disappear once the loop DNA has been fully replicated, resulting in two full-sized daughter DNA loops (E).

The close correlation observed between DNA replicon size and average DNA loop size in several types of metazoan cells strongly suggests that such DNA loops may correspond to the actual replicons in vivo [9,44]. There are several possible ways in which loop DNA may be replicated in vivo (Figure 8). The simplest mechanism that agrees with the standard text-book description of DNA synthesis is the tracking model in which the replication complex binds to a replication origin and from there moves along the template while synthesizing new DNA. In this case all loop-DNA sequences should maintain their original positions relative to the NM at all times during the cell cycle (Figure 8B). Hence, our results do not support such a model. On the other hand, the reel-in models for DNA replication imply that the replication machinery is fixed on the NM and the loop DNA is reeled through the replication complex while the newly synthesized DNA is extruded from the replication factory on the NM [16,20]. The simplest model of this kind suggests that the replication origin is located at the tip of the DNA loop and then it binds to the NM late in G1 before the start of the S phase [21], and so the original DNA loop becomes suddenly divided into two half-sized loops (Figure 8C). This model implies that all loop DNA sequences would shift to closer positions relative to the NM during S phase but also that the LARs would be the last sequences to be replicated. However, this model is not consistent with the evidence that so far most well-characterized origins of replication in metazoans are located next or very close to LARs [5,9] and so LARs very likely correspond to early-replicating sequences. Therefore, we may assume that NM-embedded looped DNA regions (Figure 5A) either contain or are adjacent to the potential origins that after firing establish the active replication forks that remain fixed to the NM while the template is reeled towards them. Replication factories are large enough to accommodate several contiguous DNA loops that may be replicated almost simultaneously [15,20]. Indeed, it is known that adjacent origins of replication in a mammalian chromosome often fire simultaneously and that replicon clusters that share the same replication factory stably replicate at the same stage of the S phase throughout consecutive cell cycles [15,45].

## Conclusions

The present results suggest that looped DNA moves in a sequential fashion, as if reeled in, towards the NM during DNA replication in vivo and then returns to its original position in newly quiescent cells, once the liver regeneration has been achieved, thus supporting the notion that during replication it is the DNA template that is pulled towards the replication factories on the NM. The results are consistent with a reel-in model of DNA replication in vivo in which the replication complexes are fixed in factories organized upon the NM [20,21] towards which the DNA template migrates in a progressive, sequential fashion so as to be replicated (Figure 8D) thus supporting early observations, using the same animal model, which suggested DNA replicates in vivo by moving through fixed replication complexes on the NM [16]. So far we can only speculate about the detailed molecular mechanisms involved in such a reel-in process. Nevertheless, our results provide further support to the notion that the structural DNA loops attached to the NM correspond to the actual replicons in metazoan cells.

## Methods

### Animals

Male Wistar rats weighing 200-250 g were used in accordance with the official Mexican norm for production, care and use of laboratory animals (NOM-062-ZOO-1999).

### Partial hepatectomy

Surgical removal of two-thirds of the liver (mechanical partial hepatectomy) was performed between 7 and 8 am, under ether anaesthesia [10]. Rats were sacrificed using ether anaesthesia at 24 h and 7 days after mechanical hepatectomy.

### Hepatocytes

Primary rat hepatocytes were obtained from rat livers (normal or hepatectomised), using the protocol described previously [10]. Briefly, the livers were washed in situ by perfusion with PBS without Ca^2+ ^and Mg^2+ ^(PBS-A) at 37°C for 5 min at 15 ml/min for non-hepatectomised rats and for 2 min for hepatectomised rats. The tissue was further perfused with a solution of collagenase IV, Sigma (0.025% collagenase with 0.075% of CaCl_2 _in HEPES buffer, pH 7.6) for 8 min for non-hepatectomised rats and for 3 min for hepatectomised rats. Viable hepatocytes were counted in a haemocytometer and used immediately for preparing the nucleoids (see below).

### Parameters of liver regeneration

Liver regeneration progression, including main peak of DNA synthesis and return of hepatocytes to G0 after completion of liver regeneration, was estimated by determination of thymidine kinase activity in the cytosolic liver fraction using a radiometric method as we have previously described [46].

### Preparation of nucleoids

The DNA loops plus the nuclear substructure constitute a ''nucleoid'', a very large nucleoprotein aggregate generated by gentle lysis of a cell at pH 8 in non-ionic detergent and the presence of high salt concentration. Nucleoids were prepared as described previously [12]. Briefly, freshly isolated and washed hepatocytes were suspended in ice-cold PBS-A, aliquots of 50 μl containing 3 × 10^5 ^cells were gently mixed with 150 μl of a lysis solution containing 2.6 M NaCl, 1.3 mM EDTA, 2.6 mM Tris, 0.6% Triton-X 100 (pH 8.0). After 20 min at 4°C, the mixture was washed in 14 ml of PBS-A at 4°C for 4 min at 3000 rpm (1500 g). The pellet was recovered in a volume ranging from 200 to 300 μl.

### DNase-I digestion of nucleoid samples

The washed nucleoids were pooled for setting up the DNase I digestion curves (1.8 × 10^6 ^nucleoids in 1.2 ml of PBS-A) and mixed with 5 ml of DNase I digestion buffer (10 mM MgCl2, 0.1 mM dithiothreitol, 50 mM Tris at pH 7.2). Digestions were carried out at 37°C with 0.5 U/ml DNase I (Sigma). Each digestion time point aliquot contained 3 × 10^5 ^nucleoids. Digestion reactions were stopped by adding 200 μl of stop buffer (final EDTA concentration of 30 mM). The stop buffer contains 0.2 M EDTA and 10 mM Tris at pH 7.5. After digestion with DNase I, the NM-bound DNA was determined by spectrometry on aliquots of partially digested nucleoid samples that were washed and further handled as described previously [12]. The final nucleoid pellet was re-suspended in 200 μl double distilled-H_2_O to be used directly as template for PCR.

### Genomic DNA primers

Distinct sets of primers were designed for establishing the topological positions relative to the NM of fifteen small DNA sequences located along 162 kbp of the genomic region containing four members of the rat albumin gene family [23]. Briefly, primer pairs were designed approximately each 10 kb in order to establish rather regular intervals along the region studied. All primers sets were designed with a length of 20-25 bp, G-C content between 50-55%, Tm of 55-60°C, and PCR products of 250-550 bp (Table 1). Secondary structures and dimmers/duplexes were avoided. Also, primers were designed for small amplicons (≤ 418 bp) located in the 5' ends of four non-related genes that belong to separate chromosomes territories within the nucleus (Table 5).

### PCR amplification

For most experiments ten nanograms of nuclear matrix-bound DNA were used as template for PCR. PCR was carried out using 0.7 U GoTaq DNA Polymerase (Promega), 2.5 mM MgCl_2_, 0.2 mM of each dNTP and 0.1 mM of each primer. Yet when indicated (Figure 6 Table 4) sixty nanograms of nuclear matrix-bound DNA template and 1.25 U GoTaq were used instead. Amplification was performed in an Applied Biosystems 2720 thermocycler and the same amplification program was used for all pairs of primers: initial denaturising step at 94°C for 5 min, denaturising step at 94°C for 45 s, annealing at 56°C for 30 s, and extension at 72°C for 1 min for 35 cycles, with a final extension at 72°C for 10 min. The identity of all the amplicons was confirmed by restriction analysis with the appropriate restriction enzymes. Amplicons were electrophoresed on 2% agarose gels and visualized using ethidium bromide staining (0.5 μg/ml), recorded and analyzed using a Kodak 1D Image Analysis Software 3.5 system. Amplicons were scored as positive or negative on partially digested nucleoid samples, depending on whether they are detectable by the software using the default settings.

## Authors' contributions

JCR-M carried out the experiments and analyzed the data, RH-M and FM evaluated results and suggested experiments, AA-A designed the study, analyzed the data and wrote the manuscript. All authors read and approved the final manuscript.
